# Tunable graduated filters based on electrochromic materials for spatial image control

**DOI:** 10.1038/s41598-019-52080-1

**Published:** 2019-11-01

**Authors:** Alexander Hein, Carsten Kortz, Egbert Oesterschulze

**Affiliations:** Department of Physics, Physics and Technology of Nanostructures, Technische Universität Kaiserslautern, 67663 Kaiserslautern, Germany

**Keywords:** Micro-optics, Electrochemistry

## Abstract

Passive graduated filters with fixed absorption profile are currently used in image recording to avoid overexposure. However, a whole set of filters with prescribed gradients is required to cope with changing illumination conditions. Furthermore, they demand mechanical adjustment during operation. To overcome these deficiencies we present a microfabricated active electrochromic graduated filter which combines multiple functionalities: The overall absorbance, the position of medium transmission as well as the magnitude of its gradient can be tuned continuously by electrical means. Live image control is possible using low operation voltages in the range of ±2 V to reach a high change in optical density ΔOD of 1.01 (400 nm to 780 nm) with a coloration and bleaching time 1.3 s and 0.2 s, respectively. Owing to their low volume and power consumption they are suitable for widespread applications like in smartphones, surveillance cameras or microscopes.

## Introduction

Since the very beginnings of imaging, optical filters have always been the tool of choice to deal with changing illumination conditions. For intensity control neutral density (ND) filters are most often used to reduce the amount of incident light which have proven to be suitable to avoid overexposure. Along with classic passive filter elements, active filter devices with tunable homogeneous absorption have been developed which rely on the electrochromic (EC) effect. These EC devices consist of an electrochemical cell with two electrodes and an electrolyte in between, while EC batteries utilize an EC film as the cathode and a metal foil as the anode^1^. In both cases, the incorporated EC materials undergo reversible redox processes to adjust their absorption^2–8^.

Up to now, these active filters have been commercially exploited in smart windows and automatically dimming rear-view mirrors as homogeneous light shutters and have also proven to be suitable for a miniaturized EC iris^9–13^. However, in most applications for imaging systems like smartphones, surveillance cameras or microscopes, high brightness differences may occur in the scene. Therefore, graduated filters were introduced which darken only the overexposed parts of the image. In this way the brightness of the scene is adapted to the dynamic range of the camera enhancing the overall image contrast.

As an alternative to the use of graduated filters, digital image postprocessing has become popular and convenient as it generates High Dynamic Range (HDR) images by blending several images with different exposures^14,15^. However, this technique may cause errors when moving objects come into play, is time consuming, and demands excessive computing power. Capturing an image with a graduated filter eliminates these disadvantages as only a single image is taken.

**Figure 1:**
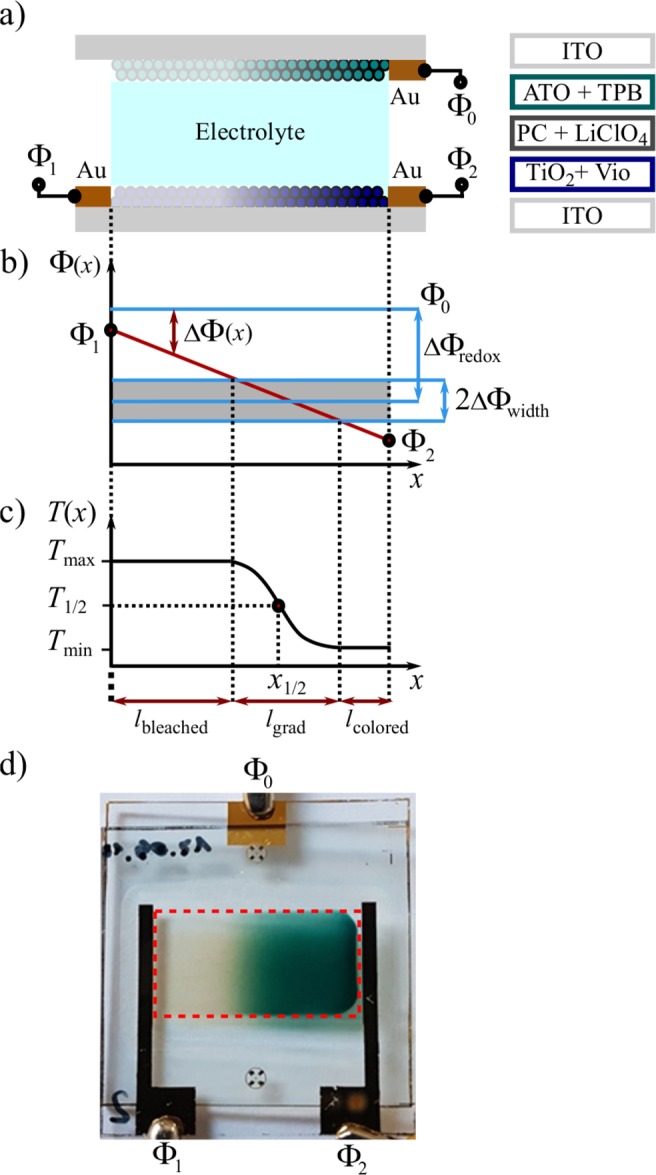
Schematic cross-section of our device including the materials involved in the device (**a**) as well as a linear potential distribution (**b**) and the resultant transmission distribution. (**c**) Optical image of a working graduated filter (**d**) with ${\Phi }_{1}=0.0\,{\rm{V}}$ and ${\Phi }_{2}=-\,1.7\,{\rm{V}}$ resembling the schemes in (**b**, **c**). The dimension of the optical active area indicated by the red dotted frame is 20 × 10 mm^2^.

Passive graduated filters consist of a glass sheet half coated with an optically absorbing absorption layer of predefined transmission *T*_min_. The layer exhibits a soft edge where its transmission increases continuously to the transparent state over a distance *l*_grad_ (see Fig. 1(c)). Both the transmission *T*_min_ as well as the magnitude of the transmission gradient $({T}_{{\rm{\max }}}-{T}_{{\rm{\min }}})/{l}_{{\rm{grad}}}$ are fixed key parameters of a passive graduated filter. Changing illumination conditions therefore require a vast assortment of graduated filters with different predefined transmission and gradients. This leads us to the major drawbacks of using these passive graduated filters: their fixed lateral transmission course, the inconvenience regarding both mechanical handling and adjustment with respect to the given light distribution, and their storage.

We now present an active graduated filter that overcomes these deficiencies as its overall transmission, the position ${x}_{\mathrm{1/2}}$ of medium transmission and the gradient’s magnitude $({T}_{{\rm{\max }}}-{T}_{{\rm{\min }}})/{l}_{{\rm{grad}}}$ can be electrically tuned. Our basic idea is to extend the functionality of well-established homogeneously tunable EC filters by applying a potential difference along the working electrode rather than a single potential. In this way a spatially varying potential distribution is established which defines the continuous coloration gradient by virtue of the EC materials.

## Results and Discussion

### Working principle

The schematic setup of our graduated EC device is shown in Fig. 1(a). Two transparent conductive oxide (TCO) coated glass substrates carry the EC electrodes responsible for coloration of the device. We chose indium tin oxide (ITO) as TCO. The electrolyte in between completes the electrochemical cell. The detailed composition of our chosen EC materials will be discussed below. During operation the upper electrode, i.e. the counter electrode, is connected to the potential ${\Phi }_{0}$. In contrast to the set-up of conventional EC devices, we have applied a potential difference ${\Phi }_{1}-{\Phi }_{2}$ to the working electrode (see Fig. 1(b)). Owing to the ohmic resistance of the ITO layer, we yield a linear spatial variation of the potential $\Phi (x)$ across the working electrode. For the electrochromic coloration at position *x* the potential difference $\Delta \Phi (x)=\Phi (x)-{\Phi }_{0}$ between the counter and working electrode is decisive. This potential difference $\Delta \Phi (x)$ determines the local redox state of the EC materials and creates the desired coloration. To illustrate the working principle, Fig. 1(b) shows a prescribed potential gradient on the working electrode causing the transmission characteristics in Fig. 1(c) with the areas of homogeneous high and low transmission on the left and right side, respectively, and the desired graduated transmission in between. In the following we introduce a theoretical model to understand the link between the electrochemical actuation and the resultant optical device response.

The level of coloration at location *x* on the EC electrodes is defined by the concentration of reduced and oxidized molecules depending on the electrochemical potential $\Delta \Phi (x)$. The steady state of a reversible redox process is generally described by the Nernst equation

1 $$\Delta \Phi (x)={\Delta \Phi }_{{\rm{redox}}}-\frac{RT}{zF}\cdot \,\mathrm{ln}(\frac{{c}_{{\rm{red}}}(x)}{{c}_{{\rm{ox}}}(x)}),$$

where *R* is the universal gas constant, *T* the ambient temperature, *z* the number of electrons transferred in the reaction, *F* the Faraday constant and ${\Delta \Phi }_{{\rm{redox}}}$ the standard electrochemical redox potential of the EC material. In case the EC material is colored in its reduced state we can substitute ${c}_{{\rm{red}}}={c}_{{\rm{colored}}}$. This requires that all other EC molecules are oxidized and therefore in their bleached state ${c}_{{\rm{ox}}}={c}_{{\rm{bleached}}}=1-{c}_{{\rm{colored}}}$. Thus by replacing *c*_ox_ in Eq. (1) we obtain:

2 $${c}_{{\rm{colored}}}(\Delta \Phi (x))=\frac{1}{1+\exp (\frac{zF}{RT}(\Delta \Phi (x)-{\Delta \Phi }_{{\rm{redox}}}))}\mathrm{.}$$

We receive a sigmoidal dependence of the percentage of colored EC molecules ${c}_{{\rm{colored}}}$ on the local potential $\Delta \Phi (x)$. Furthermore, ${c}_{{\rm{colored}}}(\Delta \Phi (x))$ can be related to the transmitted intensity ${I}_{{\rm{trans}}}(x)$ exploiting the Beer-Lambert law of optical absorption

3 $$\mathrm{ln}(\frac{{I}_{{\rm{trans}}}(x)}{{I}_{{\rm{0}}}})=-\,\varepsilon \cdot d\cdot {c}_{{\rm{colored}}}(\Delta \Phi (x)),$$

where $\varepsilon$ is the characteristic optical extinction coefficient and *d* the thickness of the EC material. By entering ${c}_{{\rm{colored}}}$ from Eq. (2) and solving for the transmitted intensity ${I}_{{\rm{trans}}}(x)$, we yield:

4 $${I}_{{\rm{trans}}}(x)={I}_{0}\cdot \exp (\frac{-\varepsilon \cdot d}{1+\exp (\frac{zF}{RT}(\Delta \Phi (x)-{\Delta \Phi }_{{\rm{redox}}}))}).$$

Thus a linear variation of the potential $\Delta \Phi (x)$ along the working electrode, i.e. a constant potential gradient, results in a non-linear, rather sigmoidal spatial transmission distribution ${I}_{{\rm{trans}}}(x)$ of our device with the redox potential ${\Delta \Phi }_{{\rm{redox}}}$ of the EC molecules and the tunable magnitude of the potential gradient as key determinants. Due to the Nernst Eq. (1) a change of coloration is thus only obtained in the potential range ${\Delta \Phi }_{{\rm{redox}}}\pm \Delta {\Phi }_{{\rm{width}}}$ of the involved redox reactions of our EC molecules. This potential interval is indicated in Fig. 1(b) by the gray bar. If $\Delta \Phi (x)$ does not reach this gray area ($|\Delta \Phi (x)| < |{\Delta \Phi }_{{\rm{redox}}}|-|{\Delta \Phi }_{{\rm{width}}}|$), all EC molecules are in their bleached state according to Eq. (2) yielding the transparent area with width ${l}_{{\rm{bleached}}}$. On the other hand, saturation of coloration is reached for $|\Delta \Phi (x)| > |{\Delta \Phi }_{{\rm{redox}}}|+|{\Delta \Phi }_{{\rm{width}}}|$, i.e. $\Delta \Phi (x)$ exceeds the gray bar, leading to the dark area of width ${l}_{{\rm{colored}}}$. By varying the external potentials ${\Phi }_{1}$ and ${\Phi }_{2}$, we control the linear potential drop crossing the gray bar and thus have the freedom to translate *x*_1/2_ and adapt the magnitude of the gradient by means of adjusting ${l}_{{\rm{grad}}}$.

So far we have solely discussed the redox behavior of the working electrode. But, in our graduated filter device we use complementary EC molecules on the working and counter electrode. Most interesting the prescribed absorption gradient on the working electrode is transferred concurrently to the counter electrode. In this way we receive at any location *x* of the device the same almost neutral spectral absorption whose amount is controlled by the local potential difference $\Delta \Phi (x)$. To explain this behavior we have to consider the charge conservation during the redox reaction: If we locally initiate reduction of the EC material on the working electrode due to the given $\Delta \Phi (x)$ at this point, an equal amount of charge is used for oxidation of the complementary EC molecules at the same position *x* on the counter electrode.

A proof of principle is given in Fig. 1(d) showing an optical image of our graduated filter device in a switched state demonstrating the achievable contrast and magnitude of the transmission gradient. While the left side of the filter is clear, the right side shows the desired dark coloration with the graduated area in between in accordance to the schemes in Fig. 1(b,c).

### Electrochemical characterization

Based on preliminary work in literature, the chosen complementary EC materials were tetraphenylbenzidine (TPB) on the counter electrode (dimerized from triarylamine, see Supplementary Information) and viologen on the working electrode^13^. By means of an attached phoshonate acid group, they were chemically adsorbed onto the surface of an antimony doped tin oxide nanoparticle layer (ATO NPL) and a TiO_2_ NPL, respectively^16,17^. The nanoparticles were chosen to provide high conductivity in the potential range matching the redox potential of its assigned EC material^18^. Additionally, they provide a high specific surface area accommodating a large number of EC molecules and therefore enable high contrast. Our device therefore benefits from both the fast response of the organic EC molecules and the high conductivity and porosity of the inorganic metal oxide nanoparticles.

**Figure 2:**
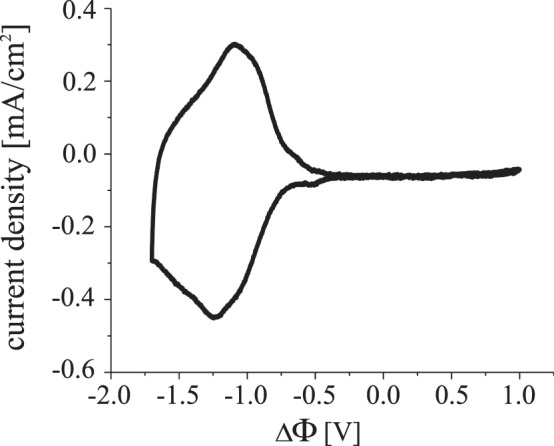
Cyclic voltammogram of the device obtained with a scan rate of 50 mV/s. For homogeneous coloration, we used $\Delta \Phi ={\Phi }_{1}-{\Phi }_{0}$ with ${\Phi }_{1}={\Phi }_{2}$.

For the electrochemical characterization of the encapsulated device we recorded the cyclic voltammogramm (CV) for homogeneous coloration with ${\Phi }_{1}={\Phi }_{2}$ and thus $\Delta \Phi ={\Phi }_{1}-{\Phi }_{0}$ (see Fig. 2). In this two electrode setup, the counter electrode also served as reference electrode.

The CV shows one broad redox peak from −0.6 V to −1.8 V for coloration and bleaching, respectively, which corresponds to the schematically drawn gray bar in Fig. 1(b) representing the potential range of coloration change. It is important to note that the CV shows the behavior of both viologen and TPB in a superposition as the application of a negative potential to the working electrode results in the simultaneous reduction of viologen on the working electrode and oxidation of TPB on the counter electrode (for details see Supplementary Information). The fact that only one strong redox peak occurs for coloration and bleaching, respectively, proves that the coloration and bleaching of viologen and TPB happen concurrently at the same potential regime. This is imperative when creating a graduated filter.

### Spectral characterization

**Figure 3:**
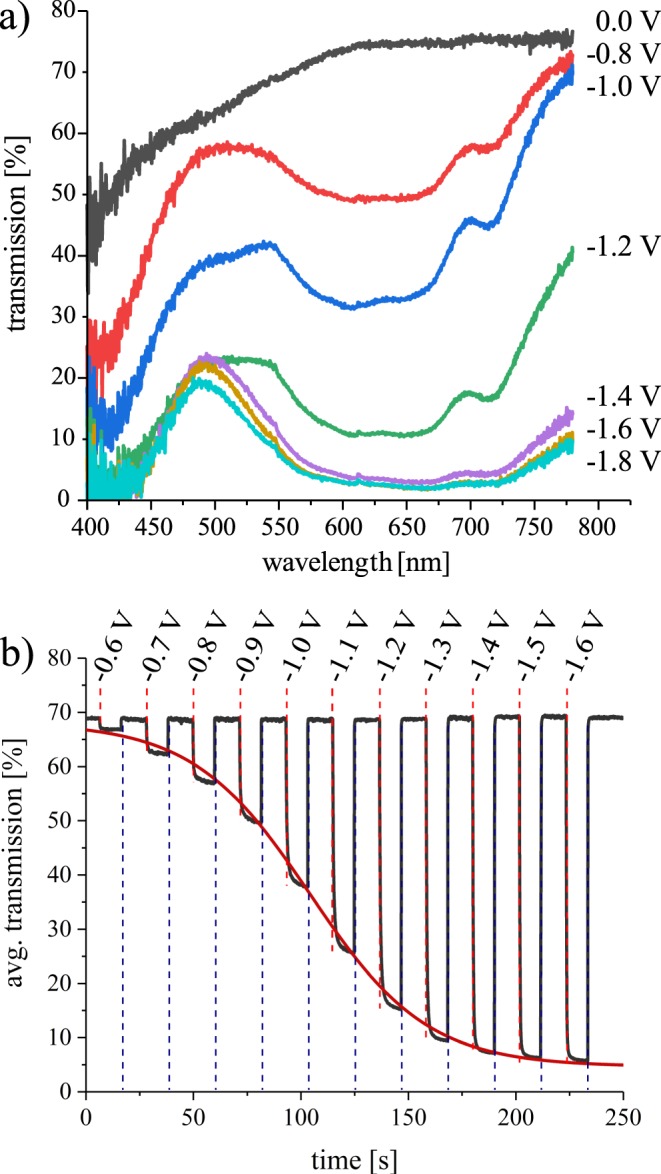
Spectral transmission (**a**) and temporal behavior (**b**) for different potentials $\Delta \Phi$ applied. For the average transmission, the wavelength range from 400 nm to 780 nm was considered. Switching in (**b**) was carried out by applying first the denoted potential (red dotted line) and then +1.5 V (blue dotted line) for 10 s each. In accordance to Eq. (4), the sigmoidal red curve was fitted to the transmission values in the colored steady state and reaches 99.9% correlation.

Spectroelectrochemical measurements were conducted to verify the potential dependent sigmoidal transmission response. Figure 3(a) shows the spectral transmission of the homogeneously colored cell (i.e. ${\Phi }_{1}={\Phi }_{2}$) for varying potential $\Delta \Phi ={\Phi }_{1}-{\Phi }_{0}$. Each spectrum corresponds to an equilibrium state and is stable without further coloration or bleaching even after removing the voltage source. We observed that this “memory effect”^11,19,20^ is strong for our cell, so we still have 75% of the initial transmission change left 30 minutes after removing the potential.

In the transparent state, the average transmission in the wavelength range from 400 nm to 780 nm is 68.3%. When a negative $\Delta \Phi$ is applied, the transmission starts to drop almost uniformly for each wavelength. This confirms the favorable behavior supposed before: The coloration of viologen and TPB happening in between −0.6 V and −1.8 V complement each other in the desired way so we keep the almost neutral color when passing this range. The use of complementary molecules further significantly increases the achievable contrast and promotes coloration over a broad spectral range as was proven by the comparison with a cell without TPB (see Supplementary Information).

For the calculation of the coloration efficiency CE, we integrated the current during coloration in the cyclic voltammogramm in Fig. 2 and the corresponding transmission in Fig. 3(a) in the transparent and opaque state, respectively. At a wavelength of 650 nm, we reach a value of CE = 280 mC/cm^2^, which slightly exceeds the CE of other EC systems using viologen molecules^21–23^.

Figure 3(b) illustrates the temporal transmission response when applying rectangular voltage pulses to the filter device. A sigmoidal fit function according to Eq. (4) accurately describes the homogeneously switched transmission $I(\Delta \Phi )$ at each potential $\Delta \Phi$ and again proves the concurrent coloration of TPB and viologen (for parameters see Supplementary Information). We observed an average coloration time $\overline{{t}_{{\rm{c}}}}=1.3\,{\rm{s}}$ and a bleaching time $\overline{{t}_{{\rm{b}}}}=0.2\,{\rm{s}}$ (switching time defined as 90% of the transmission change) which is an excellent result in comparison to literature^20–22,24,25^. When switching from 0 V to −1.6 V, we reach an average Michelson contrast of 82.1%, which corresponds to an optical density of 1.01. Commercial graduated filters are offered in 1 F stop, 2 F stop or 3 F stop filters with the latter having the highest optical density of 0.9. The tunable range of our device therefore outperforms such a set of filters and combines them in a single device. Data concerning the cyclic stability of the device is provided in the Supplementary Information, showing that after 500 cycles, 86% of the initial transmission change is maintained.

### Graduated filters

**Figure 4:**
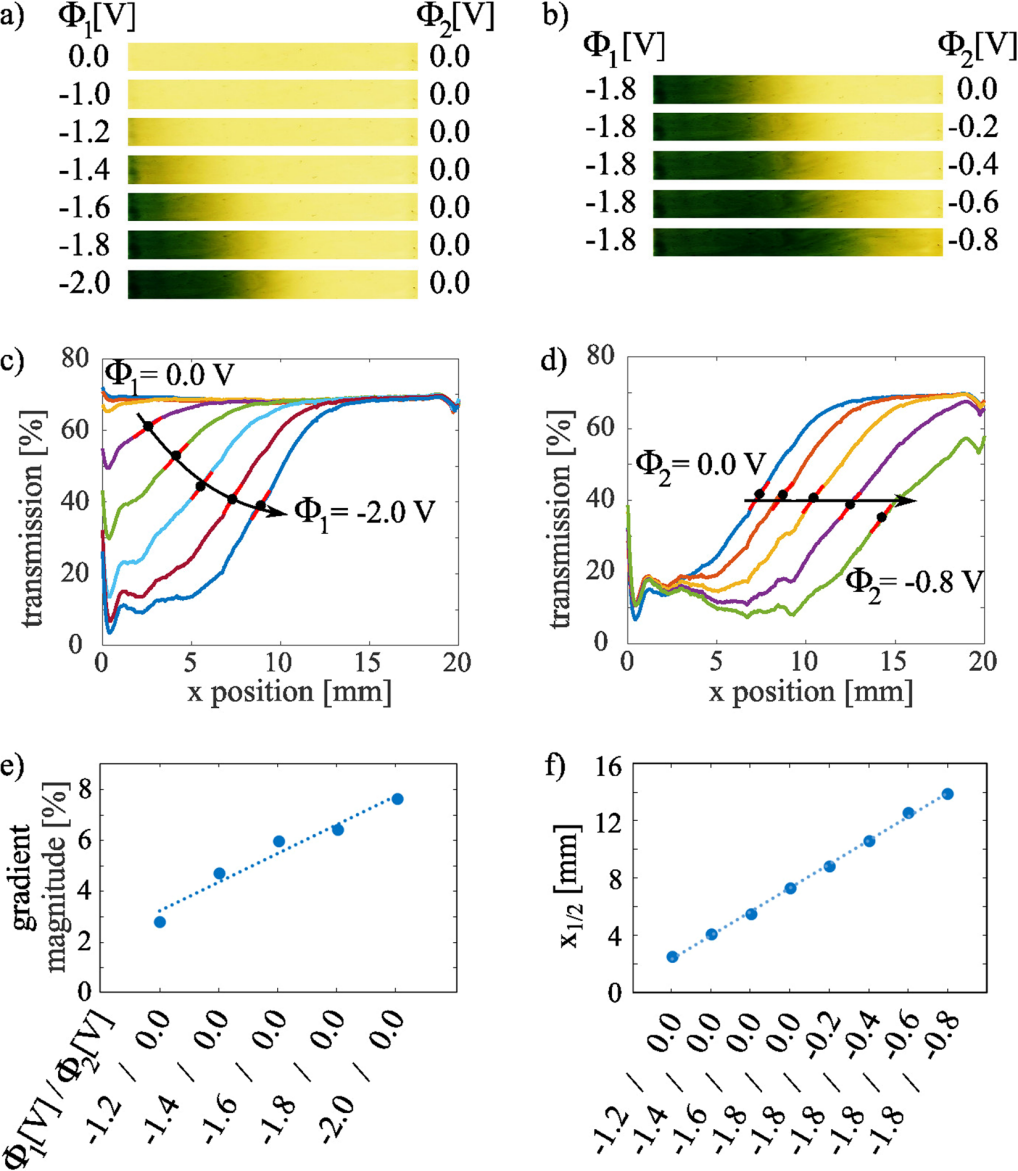
Image details of the filter (**a**,**b**) and corresponding graduated slopes for the indicated potential combinations ${\Phi }_{1}$ and ${\Phi }_{2}$ in (**c**,**d**). A red line in the graduated slopes indicates the gradient’s magnitude at its center position *x*_1/2_ marked by a black dot. Arrows were added to indicate the translation of *x*_1/2_. (**e**,**f**) Show the magnitude and *x*_1/2_ for the given potential combinations. Each step in (**f**) moves *x*_1/2_ by 1.65 mm.

We now demonstrate the performance of the graduated filter device by showing multiple graduated profiles which were recorded applying different combinations of potentials ${\Phi }_{1}$ and ${\Phi }_{2}$ to the working electrode following the scheme presented in Fig. 1(b,c). Figure 4 shows the image details and the spatial transmission for the indicated applied potentials. In Fig. 4(a,c), we created a rising local absorption on the left side by applying a negative potential ${\Phi }_{1}$ and decreasing it in steps of −0.2 V while keeping ${\Phi }_{2}=0$ fixed. Each of these profiles is temporally stable and switching happens with the same time constants as described above for homogeneous switching (see video in Supplementary Information). According to the aforementioned sigmoidal dependence of the transmission according to Eq. (4) on the local potential $\Delta \Phi (x)$, we now observe a sigmoidal spatial transmission distribution because of the lateral linear potential drop (for details see Supplementary Information). Moreover, the profiles clearly show that the right side with the applied potential of ${\Phi }_{2}=0.0\,{\rm{V}}$ stays in the transparent state while the left side becomes darker. This is a proof of the working principle of the tunable graduated filter claimed before: The graduated coloration profile is established on both electrodes although the potential gradient is only applied to the working electrode.

To investigate the symmetry of our device, we swapped the potentials displayed in Fig. 4(a) (see Supplementary Information). Quantitative evaluation of the symmetry was done by determining the correlation of these results to the vertically flipped profiles in Fig. 4(c). We achieved values for the coefficient of determination from 98.2% up to 99.0%, which again confirms the feasibility of the working principle as well as the accuracy of the microfabrication route. The flipping of the EC filter takes only 1.3 s and conveniently replaces the manual rotation of a commercially available passive graduated filter.

In Fig. 4(c), the position *x*_1/2_ of medium transmission, i.e. the point of 50% transmission change, is indicated by a black dot and the slope of the associated red line marks the gradient’s magnitude at this point. By applying an increasingly negative potential to the left side, the gradient’s magnitude is increased from 2.8%/mm at −1.2 V to 7.6%/mm at −2.0 V as shown in Fig. 4(e). This is accompanied by a translation of *x*_1/2_, which is indicated by the arrow in Fig. 4(c). Corresponding to Fig. 1, this behavior can be explained by the increasingly negative potentials which expand to the middle part of the filter.

For further translation of *x*_1/2_, we kept the left side in the colored state applying a fixed potential ${\Phi }_{1}=-\,1.8\,{\rm{V}}$ and applied increasingly negative potentials to the right. The results are shown in Fig. 4(b,d), again with high symmetry for swapped potentials (Supplementary Information). In accordance to the behavior of the homogeneously colored EC filter in Fig. 3(b), the coloration on the right side only occurs for potentials lower than −0.6 V. The profiles in Fig. 4(d) further show that translation of *x*_1/2_ is possible without changing the gradient’s magnitude: Each potential step shown in Fig. 4(f) successively moves *x*_1/2_ further to the right with an almost linear behavior for the given voltage combinations (coefficient of determination R^2^ = 99.9%). Overall, *x*_1/2_ can be shifted by 11.4 mm which is more than half the 20 mm filter width.

**Figure 5:**
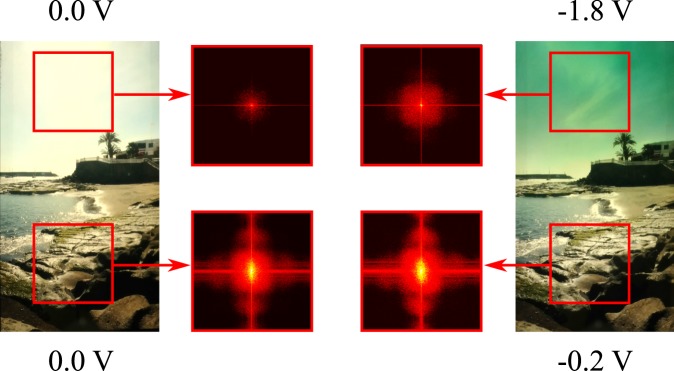
Images taken of a landscape scene with the graduated filter device placed in front of the camera lens in vertical direction. The given potentials were applied to the top and bottom of the EC filter device creating the transparent state (left) and a graduated state (right). Both images were taken with the same exposure parameters. The additional images show Fourier transformations of the framed areas in the images to identify the amount of transmitted spatial frequencies.

This favorable behavior was exploited in a realistic application shown in Fig. 5. We used a commercial camera and positioned our graduated filter device in front of the lens, while the landscape scenery was provided by a display. The good optical quality of our device reveals that scattering due to the immersed nanoparticles of 20 nm diameter is negligible. In the left image, the illumination conditions cause an overexposure of the bright sky, while the foreground underneath is imaged with appropriate contrast. This is also shown by the attached Fourier transformations of top and bottom parts of the image marked by the red rectangles. We now use the tunable graduated filter to darken only the overexposed upper part of the image and precisely adjust *x*_1/2_ of the transmission profile to the horizon line of the scene image. The Fourier transformation reveals that more spatial frequencies (i.e. details in the clouds) become visible in the upper part of the image while the lower part with appropriate contrast stays unaffected.

During operation in the graduated state of the filter, we observed a maximum current of 20 mA between the contact pads on the working electrode with ${\Phi }_{1}=-\,2\,{\rm{V}}$ and ${\Phi }_{1}=-\,0\,{\rm{V}}$. The maximum power dissipation is therefore in the range of 40 mW. Up to now, we have not observed any effect of heating on the EC performance. A video of the switching process from the transparent to the graduated state is added to the Supplementary Information. It gives an impression of the fast adaption of the filter and its applicability in commercial imaging systems in the near future. As the working principle and the process route are not restricted to the dimensions of our filter, the utilization in miniaturized systems such as surveillance cameras or smartphones as well as in large scale devices like smart windows is feasible.

## Conclusion

We have presented an EC graduated filter to control image contrast in one-dimension. Its working principle relies on the idea to create a spatial coloration gradient applying a linear potential drop on the working electrode of our EC filter. We showed that owing to the choice of electrochemically complementary EC molecules, the graduated transmission is established on both the working and the counter electrode and follows a sigmoidal function. This behavior could be accurately predicted by our theoretical model. It was confirmed by spectroelectrochemical measurements. We reached a high average optical density of 1.01 in the optical spectral range and short switching times of 1.3 s while being able to continuously tune the gradient’s position, its magnitude and the overall absorption. The capability of the EC graduated filter was demonstrated installing it to a commercial camera and investigating its impact on the image contrast enhancement.

## Methods

Our process route described hereafter allows a reproducible, reliable microfabrication of graduated filter devices with an active optical area of 20 × 10 mm^2^ and a thickness of 55 *μ*m excluding that of the glass substrates. Manufacturing was done under clean room conditions. In Fig. 1(d), the active optical area appears clear, which can be ascribed to the high transparency and negligible scattering of the TiO_2_ nanoparticles (Ti-Nanoxide T/SP, Solaronix SA, Switzerland) and ATO nanoparticles ($\varnothing  < 20\,{\rm{nm}}$).

As substrates, we used commercially available ITO coated glass slides with $8\,\Omega /\square$ sheet resistance. For fabrication, we performed microstructuring based on UV lithography. Gold was deposited via sputter deposition and ITO was structured using hydrochloric acid for wet chemical etching. After that, the TiO_2_ and ATO nanoparticle pastes were applied using a stencil printer and calcinated at 450 °C in a vacuum oven. For heating, we used a slow ramp of 10 °C/min, a holding time of 2 h and allowed the oven to cool down naturally. We yielded TiO_2_ and ATO layer thicknesses of 10 *μ*m and 3 *μ*m, respectively. For chemical adsorption of the EC molecules, the electrodes were immersed in a solution of 1 mM TAA in ethyl alcohol or 1 mM viologen in ethyl alcohol for 15 hours, respectively. By structuring a dry film resist (Ordyl SY 300, Elga Europe s.r.l., Italy), we formed two cavities for the electrolyte (1 mol/l LiClO_4_ solved in propylene carbonate) and a surrounding UV resin, which were precisely dosed using a dispensing robot. The thickness of the cavity was hereby defined by the thickness of the Ordyl spacer layer of 55 *μ*m. Encapsulation was performed in a Ar glovebox which maintained inert Ar gas atmosphere to prevent the EC molecules from being irreversibly damaged. By joining the substrates with a displacement of 3 mm, convenient contacting of the exposed gold pads is possible as seen in Fig. 1(d).

For electrochemical characterization, we used two potentiostats “Reference 600” from Gamry Instruments to individually adjust the potentials on the working electrode. The setup for spectroelectrochemical characterization consisted of a white light source “Schott KL 1500”, several lenses for focusing and a spectrometer “Flame” from Ocean Optics. Images were taken with a Samsung camera “NX300M” with a 18–55 mm f3.5–5.6 OIS III Zoom Lens while homogenous illumination was obtained by a OLED panel (“seelectorLux100”, hema electronic GmbH, Germany). The camera was first calibrated by taking images of filters with known transmission. Hence, local transmission of the graduated EC filter could be calculated from the RGB data obtained from the images.

## Supplementary information

Supplementary Information

## Data Availability

The data of this study are available from the corresponding author upon reasonable request.
