# Supplementary material for: Tunable graduated filters based on electrochromic materials for spatial image control
Source: Sci Rep. 2019 Nov 1;9:15822. doi: 10.1038/s41598-019-52080-1 (PMC6825214; doi:10.1038/s41598-019-52080-1)
Supplement: Supplementary file 1 — Supplementary Information [file 41598_2019_52080_MOESM1_ESM.pdf]

Supplementary Material

Tunable graduated filters based on electrochromic materials for spatial image control

Alexander Hein, Carsten Kortz, Egbert Oesterschulze

Additional Information on the electrochromic materials

The electrochromic (EC) molecule adsorbed on antimony tin oxide (ATO) nanoparticles was triarylamine (TAA) which was equipped with a phosphonate acid as anchor group. However, during the first potential cycle in the range of [1 V ... -1.75 V], we observed that dimer formation occurs which generates tetraphenylbenzinide (TPB). This process has been studied in detail in the past and was found to be prevalent for triphenylamine itself and also for many substituted triphenylamines in which one or more phenyl groups were replaced [1–3]. The reaction cascade proposed in Fig. S1 is in accordance to the concepts stated in literature and describes an electron transfer - chemical reaction - electron transfer type. The initially adsorbed TAA is electrochemically oxidized when applying positive potentials and forms an unstable monocation radical TAA+·. These cations rapidly react to form TPB, therefore two TAA molecules are consumed for one TPB molecule. Two protons (indicated by -2 H^+ in Fig. S1) are lost during this reaction. However, TPB is oxidized much easier and at lower potentials than TAA which means that it undergoes further oxidation at the applied potential. This leads to the additional loss of two electrons as seen in Fig. S1 and TPB reaches the dicationic state TPB++ along with a gray coloration. When the voltage is further reduced, TPB is reduced in two steps, first reaching the monocationic state with red coloration and then the neutral transparent state.

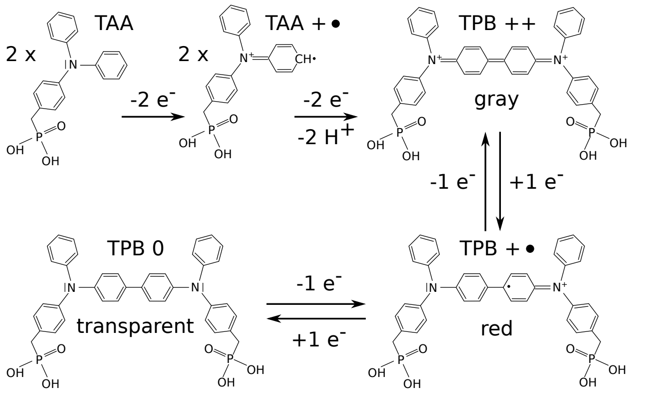

Figure S1: Reaction cascade of triarylamine (TAA) to tetraphenylbenzidine (TPB) and the subsequent reversible redox reactions of TPB with indicated coloration.

Seo et al already observed that the cyclic voltammogramms of TAA and TPB are utterly different and serve as clear indicator for this dimerization process [1]. This is because TAA is oxidized at much higher potentials than TPB which could easily be derived from the position of the oxidation peak. Additionally, they observed that the charges released when undergoing the reaction cascade from TAA to TPB were different from the charges used for reversible cycling of TPB and therefore, the current density and thus the redox peaks would be much higher. This fits the reaction cascade in Fig. S1: The conversion of TPA to TPB++ is a four electron process while the TPB++ consumes only two electrons when reacting to TPB. This assumption was proven by reverse current chronopotentiometry with a ratio of 2.0 for forward and reverse transition times.
To investigate our EC system, we conducted cyclic measurements of the first switching of our device (see Fig. S2). As we had an encapsulated device, we worked in a two electrode system and we have to consider that we do not only see the electrochemical behavior of TAA on the counter electrode but the superposition with the response of viologen on the working electrode. However, our cyclic voltammogramm shows the

identical behavior to the ones known in the literature [1, 2, 4]: The first cycle is significantly different to the others as the redox process starts at more negative potentials (-1.0 V instead of -0.6 V) and its peak reaches a much higher current density than any of the redox peaks afterwards. This is in complete agreement with the explanations above and proves the suggested reaction cascade in Fig. S1. All following cycles show the behavior typical for TPB and have an identical shape. Thus, TPB formation is almost finished after the first cycle.

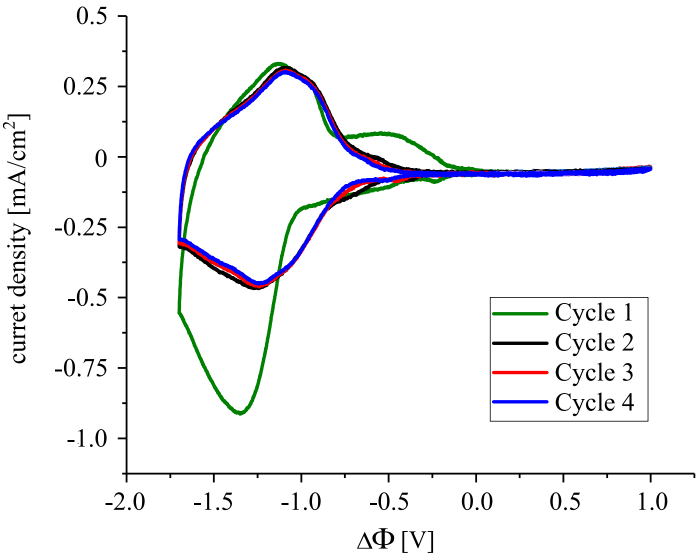

Figure S2: Cyclic voltammogramm (CV) of the device in a two electrode setup (scan rate of 50 mV/s).

When further investigating the subsequent cycles, we have already stated that the redox processes of the two EC molecules viologen on the working electrode and TPB on the counter electrode complement each other in a favorable way that only one redox peak is observed for coloration and bleaching, respectively. This allows the description of the whole device assuming only one single coloration process as done below and the broad appearance of the redox peak from -0.6 V to -1.8 V allows precise tuning of the amount of coloration. Furthermore, the CV shows that the redox peaks for coloration and bleaching have a similar shape and and appear at similar potentials although the scan rate of the cyclic voltammogramm is comparably high (50 mV/s). This does not only indicate reliable, reversible coloration of the EC electrodes but it also indicates an immediate response to a changing potential which makes fast switching possible.

Additional experimental data on the spectral performance of our complementary cell

The use of the EC viologen molecule alone as done in literature is not sufficient to achieve neutral absorption over a broad spectral range [5–7]. We therefore introduced the aforementioned TPB as complementary EC molecule, which was chemically adsorbed on the counter electrode and is colored during oxidation. It is therefore electrochemically complementary to the viologen molecule as well as spectrally because it absorbs light in a different spectral range. The effect of adding TPB to the system is shown in Fig. S3: The lowest achievable transmission for the cell without TPB is 17.2 % (average from 400 - 780 nm) while with TPB only 5.8 % of transmission remains at a potential of -1.6 V. This can be ascribed to the significantly decreased transmission in

the wavelength range 650 - 780 nm where TPB has a high absorption in its dicationic state [8,9]. Using TPB on the counter electrode is therefore a reasonable improvement to achieve broad band absorption. It is also interesting to note that the intermediate states in Fig. S3 b) show less transmission than those in Fig. S3 a). The oxidation of TPB and therefore the additional coloring starts along with the viologen coloring at -0.6 V.

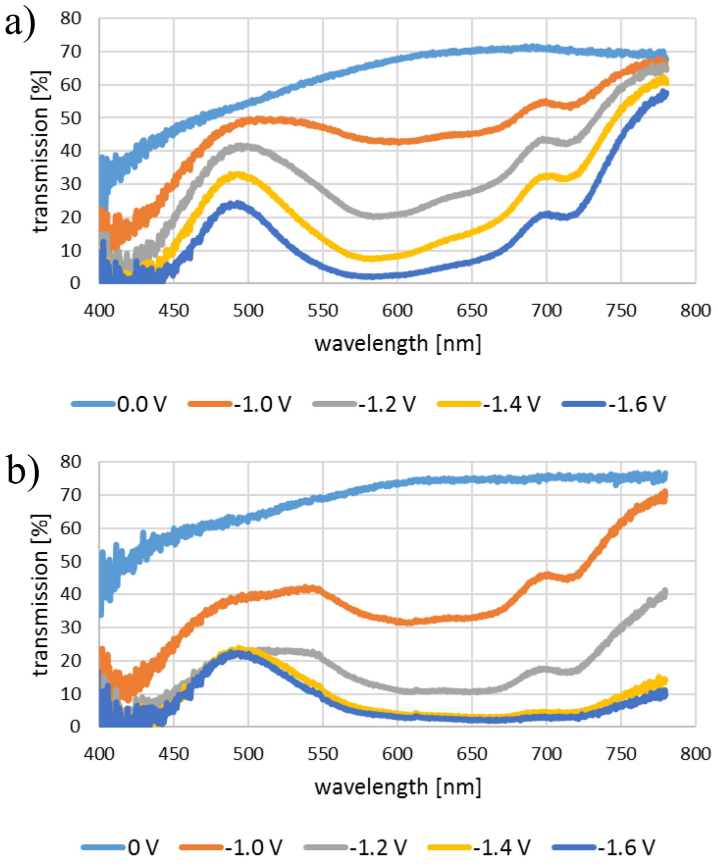

Figure S3: Transmission spectrum of a EC device without TPB (a) and with TPB (b). The applied voltages are indicated.

Additional experimental data on the potential dependent sigmoidal transmission response of the EC device

The transmission of the device can be conveniently tuned by applying a potential $\Delta\Phi$ between working and counter electrode. In accordance to our theory which relies on the Nernst equation, we use a sigmodal fit function to describe the transmission for homogeneous coloring ($\Phi_1 = \Phi_2$). Instead of describing the transmission behavior of one electrode as done in Eq. (4), we now use Eq. (S1) to describe the transmission of the whole device consisting of two EC materials on working and counter electrode. This is feasible because we have shown that the coloration on working and counter electrode

occur at the same potential, i.e. $\Delta\Phi_{\text{redox}_1} = \Delta\Phi_{\text{redox}_2}$. We therefore obtain:

$$I_{\text{trans}} = I_0 \cdot \exp\left(\frac{-\epsilon_1 \cdot d_1}{1 + \exp\left(\frac{1}{m_1}(\Delta\Phi - \Delta\Phi_{\text{redox}_1})\right)}\right) \cdot \exp\left(\frac{-\epsilon_2 \cdot d_2}{1 + \exp\left(\frac{1}{m_2}(\Delta\Phi - \Delta\Phi_{\text{redox}_2})\right)}\right)$$
$$= I_0 \cdot \exp\left(\frac{-\epsilon \cdot d}{1 + \exp\left(\frac{1}{m}(\Delta\Phi - \Delta\Phi_{\text{redox}})\right)}\right), \tag{S1}$$

with $\epsilon \cdot d = \epsilon_1 \cdot d_1 + \epsilon_2 \cdot d_2$ assuming in a simplifying approach $m \approx m_1 \approx m_2$. The data for average transmission was obtained by integrating the spectral transmission from Fig. S3.

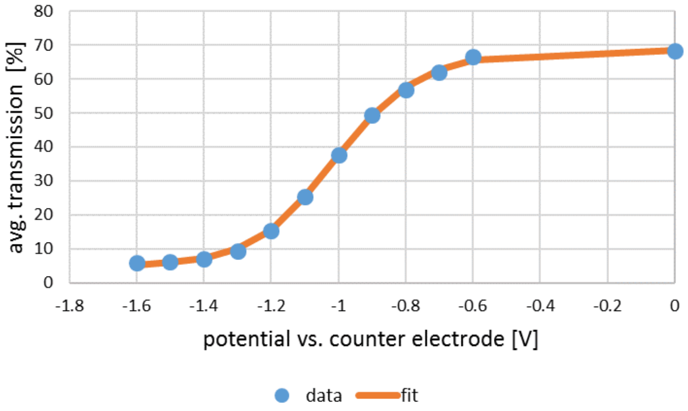

Figure S4: Average transmission (400 nm to 780 nm) of the EC device over applied potential. The sigmoidal fit done using Eq. (S1) is in excellent agreement with the data.

We therefore yield the fit parameters of Tab. S1:

Table S1: Parameters for the fit in Fig. S4

I_0	68.4 %
$\epsilon \cdot d$	2.67
Φ_{redox}	-1.17 V
m	0.14 V

and obtained an excellent correlation of 99.95 %. According to our fit function, the minimum achievable transmission is therefore 4.7 % while 90 % of the transmission change occurs in the voltage range from -0.62 V to -1.37 V. When comparing the fit parameter $\Phi_{\text{redox}} = -1.17\,\text{V}$ to the cyclic voltammogramm shown in Fig. S2, we can note that Φ_{redox} accurately matches the medium position of the reduction and oxidation peak, which is in line with common theory for cyclic voltammetry [10]. The value of m is higher than expected from theoretical derivation ($\frac{RT}{zF} \approx 26\,\text{mV}$) which is due to the fact that we are operating the cell in a two electrode setup. The indicated $\Delta\Phi$ therefore is the potential drop from counter to working electrode and not only that of a half cell. Nevertheless, the high correlation of the fit to our data is proof that the coloration of our cell can be reliably described with our derived theory.

Additional experimental data on the symmetry of the gradient for transposed potentials

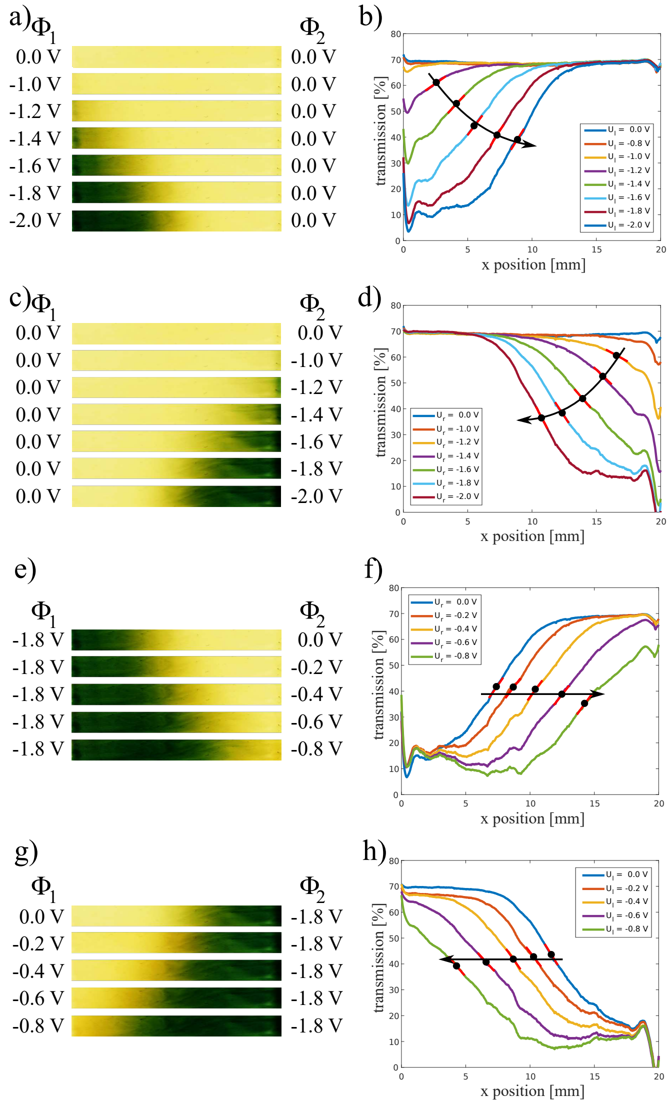

Figure S5: Image details of the filter and corresponding graduated slopes for the indicated potential combinations. The image details show the whole optical area of 20×10 mm^2, but were compressed in height by a factor of 4. The same holds for Fig. 4a) and c) in the paper. (e) - (h) show the filter in its corresponding swapped states of a) - d). The Fig. a), b), e) and f) have already been shown in Fig. 4 and are repeated for clarity.

To illustrate the working principle, we show additional images and graduated slopes of the filter in Fig. S5. We swapped the potentials thus creating a coloration on the right side while achieving the same functionality as when darkening the left side: Fig. S5 c) and d) show the tunability of the gradient's magnitude while Fig. S5 g) and h) illustrate the translation of $x_{1/2}$.
Furthermore, we vertically flipped the profiles in Fig. S5 d) and h) and determined their correlation to the profiles of Fig. S5 b) and f). The high correlation values from 98.2 % up to 99.0 % show the accuracy of our fabrication route and the symmetry of our device.

Additional information on the lateral sigmoidal transmission distribution of the EC device

By applying a potential difference to the working electrode, we create a lateral potential drop. We now aim to exploit the derived and proven sigmoidal transmission/potential relation (see Fig. S4) creating the desired lateral transmission. According to

$$I_{\text{trans}}(x) = I_0 \cdot \exp\left(\frac{-\epsilon \cdot d}{1 + \exp\left(\frac{1}{m}(\Delta\Phi(x) - \Delta\Phi_{\text{redox}})\right)}\right), \tag{S2}$$

we adapted a fit curve to the lateral transmission distributions shown in Fig. S6. We assumed a linear potential along the 20 mm wide optical area using the potentials applied. The fit parameters are shown in Tab. S2.

Table S2: Parameters for the fit in Fig. S6

I_0	70.9 %
$\epsilon \cdot d$	1.62
Φ_{redox}	-1.15 V
m	0.13 V

The high correlation of 96.5 % confirms that the lateral transmission behavior of the EC filter can in fact be described by assuming a lateral potential drop along with the derived sigmoidal coloration behavior. As claimed before, the coloration behavior of the homogeneous filter can be transferred to each position x with its local potential $\Delta\Phi(x)$. Therefore, the fit parameters of Tab. S1 and Tab. S2 are very similar.

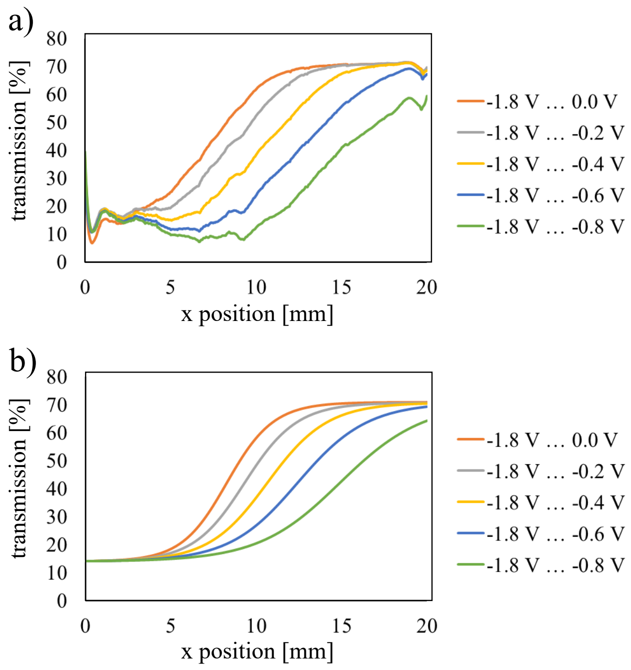

Figure S6: Data (a) and fit curves (b) of the lateral transmission for the indicated voltage combinations.

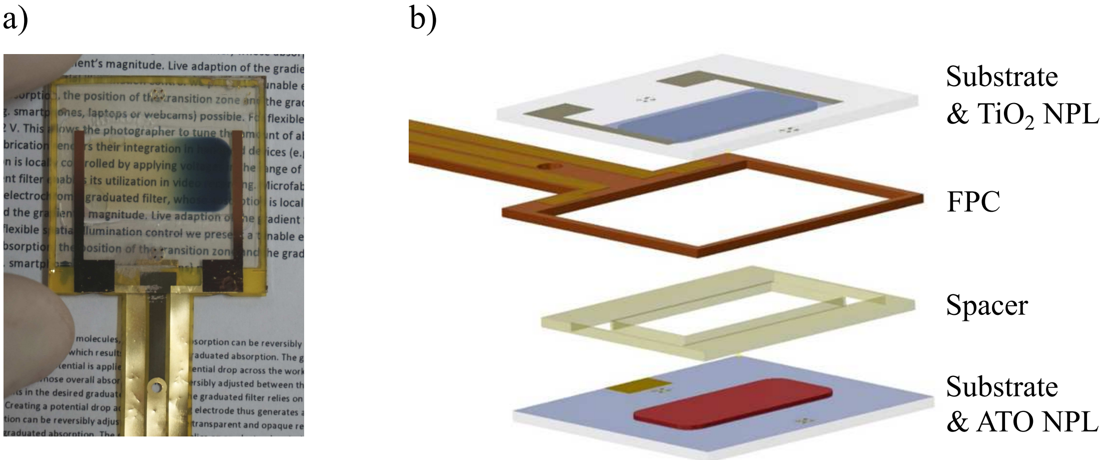

Figure S7: Image of the top view of a cell with integrated FPC (a) and schematic layered setup (b). In (a), the potential combination $\Phi_1 = 0.0\,\mathrm{V}$ and $\Phi_2 = -1.7\,\mathrm{V}$ was applied.

Additional information on the process route for integration of a flexible printed circuit (FPC)

For convenient electrical contacting, we fabricated additional devices with an integrated flexible printed circuit (FPC). These circuit boards (LeitOn Ltd., Germany) have a total thickness of only 75 µm with a thin flexible polyimide layer as support layer and structured Au coated conducting paths on the top and bottom. By the use of these FPCs, we have three advantages:

1. The substrates can be bonded without displacement, i.e. in Fig. S7 b) all Au contact pads point to the back left.

2. Only one connecting cable, the FPC is needed as we can conveniently contact the Au pads on the substrates facing each other with the FPC. The FPC has corresponding conducting paths and is sandwiched between the substrates and positioned around the spacer layer. For electric connection we apply conductive adhesive (PU 1000, Polytec PT Ltd.).

3. All electrical contacts on the FPC are located on the upside because we use a via to transfer the contact to the lower substrate in Fig. S7 b) to the upside.

Our concept was successfully applied as shown in Fig. S7 a) and encourages the application in commercial camera systems in the near future.

Additional information on the long-term stability of the filter device

To illustrate the long-term stability of our device, we applied a repeating step potential of 0 V to -1.5 V for 10 s each and recorded the device transmission. The results in Fig. S8 a) show 500 cycles. Due to the temporal compression (the experiment took 3 hours), only the maximum and minimum transmission of each cycle is visible as an envelope. To describe the long-term stability in greater detail, we extracted cycles 1 and 500 in Fig. S8 b).
During the 500 cycles of switching, we see a gradual decrease in transmission change as the minimum transmission rises. After 500 cycles 86 % of the initial transmission change

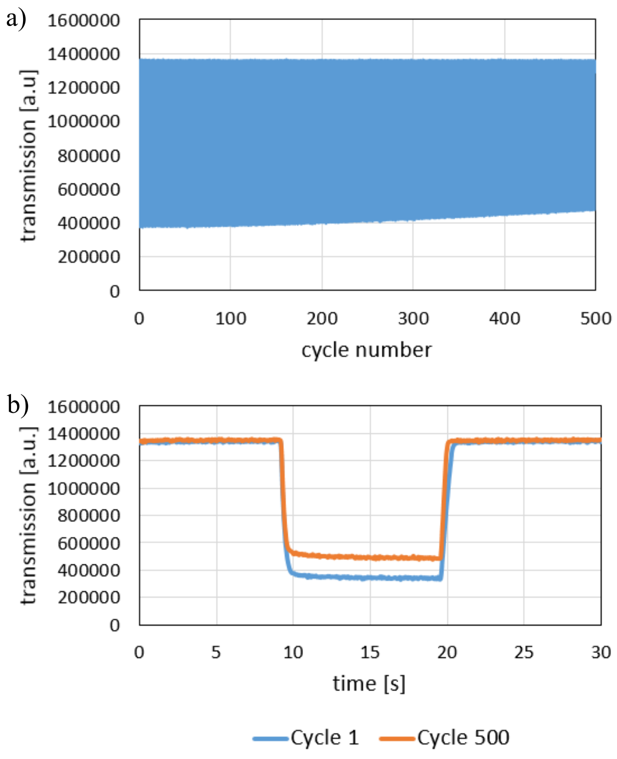

Figure S8: Transmission during cycles 1 to 500 (a) and direct comparison of cycle 1 and 500 (b).

remains. However, Fig. S8 b) shows that switching times are only slightly affected by this aging process. The coloration time is still at 0.7 s, while the bleaching times are 0.8 s and 0.5 s during cycle 1 and 500, respectively.

Additional movie "gradient.mp4"

The movie shows a fabricated EC filter device when switching from its transparent to its graduated colored state. The device was placed in a custom holder and back light illumination was performed with an OLED panel. The upper clip is connected to the counter electrode, while the two lower clips enabled to prescribe the potential gradient to the working electrode. In its transparent state, the potentials Φ_1 and Φ_2 are set to 0 V, while the graduated coloration is achieved by applying $\Phi_1 = -0.2$ V and $\Phi_2 = -1.8$ V.

References

[1] Eddie T Seo, Robert F Nelson, John M Fritsch, Lynn S Marcoux, Donald W Leedy, and Ralph N Adams. Anodic Oxidation Pathways of Aromatic Amines. *Am. Chem. Soc.*, 3618(1962):3498–3503, 1966.

[2] Robert F Nelson, Stephen W Feldberg, and Robert F Nelson. Chronoamperometric Determination of the Rate of Dimerization of Some Substituted Triphenylamine Cation Radicals. *J. Phys. Chem.*, 73(8), 1969.

[3] Kesavapillai Sreenath, Chettiyam Veettil Suneesh, Venugopal K Ratheesh Kumar, and Karical R Gopidas. Cu (II) -Mediated Generation of Triarylamine Radical

Cations and Their Dimerization. An Easy Route to Tetraarylbenzidines. *J. Org. Chem.*, 73:3245–3251, 2008.

[4] Samuel C. Creason, James Wheeler, and Robert F. Nelson. Electrochemical and Spectroscopic Studies of Cation Radicals. *J. Org. Chem.*, 37(26):4440–4446, 1972.

[5] P. Bonhôte, E. Gogniat, F. Campus, L. Walder, and M. Graetzel. Nanocrystalline electrochromic displays. *Displays*, 20:137–144, 1999.

[6] By Martin Möller, Simona Asaftei, David Corr, Michael Ryan, and Lorenz Walder. Switchable Electrochromic Images Based on a Combined Top-Down Bottom-Up Approach. *Adv. Mater.*, 16(17):1558–1562, 2004.

[7] Chil Seong Ah, Juhee Song, Seong M Cho, Tae-youb Kim, Han Na Kim, Ji Yong Oh, Hye Yong Chu, and Hojun Ryu. Double-layered Black Electrochromic Device with a Single Electrode and Long-Term Bistability. *Bull. Korean Chem. Soc.*, 36:548–552, 2015.

[8] Huan-shen Liu, Bo-cheng Pan, De-cheng Huang, Yu-ruei Kung, Chyi-ming Leu, and Guey-sheng Liou. Highly transparent to truly black electrochromic devices based on an ambipolar system of polyamides and viologen. *NPG Asia Mater.*, 9, 2017.

[9] Chil Seong Ah, Juhee Song, Seong Mok Cho, Tae-youb Kim, Hojun Ryu, and Sanghoon Cheon. Optical and Electrical Properties of Electrochromic Devices Depending on Electrolyte Concentrations and Cell Gaps. *Bull. Korean Chem. Soc.*, 37:1812–1819, 2016.

[10] C. G. Zoski. *Handbook of Electrochemistry*. Elsevier Science, 2007.
